# Dataset on the regulation of banana weevil abundance and corm damage associated with plant richness and the ground-dwelling arthropods’ food web

**DOI:** 10.1016/j.dib.2017.09.003

**Published:** 2017-09-14

**Authors:** Charlotte Poeydebat, Philippe Tixier, Luc De Lapeyre De Bellaire, Dominique Carval

**Affiliations:** a CIRAD, UPR GECO, F-97285 Le Lamentin, Martinique, France; b CIRAD, UPR GECO, F-34398 Montpellier, France; c Departamento de Agricultura y Agroforesteria, CATIE, CR-30501 Turrialba, Costa Rica

## Abstract

The data presented in this article are related to the research article entitled "Plant richness enhances banana weevil regulation in a tropical agroecosystem by affecting a multitrophic food web " [1]. It provides information about plant species richness, weevil corm damage and the abundance of different arthropod groups, including the banana weevil and its potential natural enemies and alternative preys.

Specifications Table

**Table:** 

Subject area	*Biology*
More specific subject area	*Conservation biological control*
Type of data	*Table, R script, graph*
How data was acquired	*Field survey*
Data format	*Raw, filtered and analyzed*
Experimental factors	*Abundance and damage of banana weevilCosmopolites sordidus*, *abundance of arthropod trophic groups (potential natural enemies and alternative preys ofCosmopolites sordidus*) and plant species richness
Experimental features	*Vegetation and ground-dwelling arthropod food web were monitored in plots in farmer fields across a gradient of plant richness*.
Data source location	*Reserve of Talamanca (9°00’- 9°50’ N, 82°35’ – 83°05’ W) in Costa Rica*
Data accessibility	*Data is available with the article*

**Value of the data**

• The data is valuable for other researchers working on this pest or in similar scientific field because it offers them the opportunity to compare with their own datasets and/or to independently verify or extend statistical analyses.
• This data is a contribution to the effort of the scientific community to quantify ecosystem services and in particular ecosystem services in agroecosystems associated with plant diversification.
• The data is valuable as it comes from an original experimental design using an existing plant richness gradient to study the continuous relationship between plant richness and response variable related to pest regulation service.
• This data does not focus solely on the effect of plant richness on abundance or damage of the pest, but also on potential alternative preys or predators of the pest.

## 1 Data

**Fig. 1:**
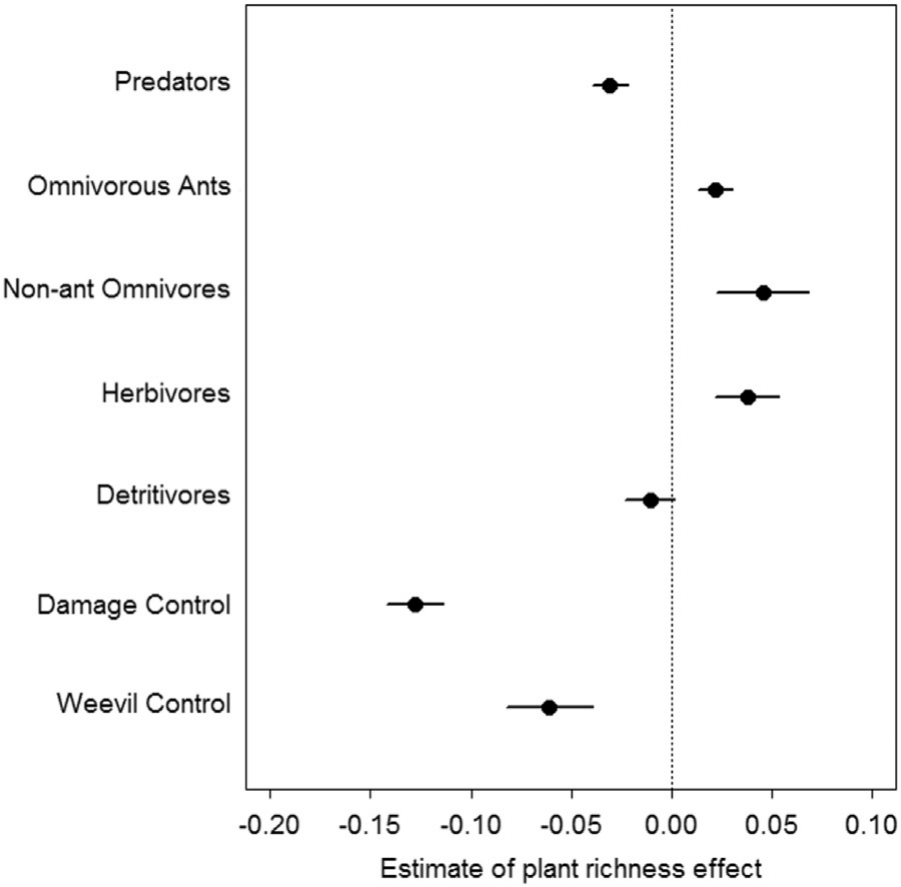
Ecosystem services related to banana weevil regulation. Effect of total plant species richness on the abundance of alternative preys, i.e. herbivores and detritivores; on the abundance of potential natural enemies, i.e. omnivores and predators; and on the adult abundance and the damage of the banana weevil. Points are GLMMs estimates (± s.e.).

The dataset presented in this article, in Table 1, comes from a fieldwork conducted in banana-based farmers’ fields in the Reserve of Talamanca in Costa Rica (9°00′–9°50′ N, 82°35′–83°05′ W) between July 2014 and January 2016. Each observation corresponds to a 10 m radius circular plot located in a field and containing a banana phytometer. Data provides information about plant species richness, corm damage measured on the phytometer, and abundances of several groups of ground-dwelling arthropods, including predators, omnivorous ants, non-ant omnivores, herbivores, detritivores and the banana pest *Cosmopolites sordidus*. In addition to the dataset, we present the outputs of statistical models that quantify the effect of total plant species richness on the abundance of each arthropod group and on corm damage. Fig. 1 displays the services or disservices associated with pest regulation provided by plant species richness, expressing the size of the effect of total plant richness on each variable.

**Table 1:** Abundances of different groups of ground-dwelling arthropods: herbivores, detritivores, non-ant omnivores, omnivorous ants, predators and banana weevils; banana weevil corm damage score; and total plant richness within the plot. Plant species richness is the sum of the low-stratum plant species richness (plants below 1.5 m high) and the high-stratum plant species richness (plants above 1.5 m high).

**Plot ID**	**Abundance**						**Corm damage score**	**Total plant richness**
	**Herbivores**	**Detritivores**	**Non-ant omnivores**	**Omnivorous ants**	**Predators**	**Banana weevils**		
CR_P1_B1	0	3	0	13	2	0	0	20.5
CR_P1_B2	0	2	5	5	1	0	0	16.5
CR_P1_B3	2	0	1	2	2	0	5	18.0
CR_P1_B4	5	2	2	0	4	1	NA	23.0
CR_P1_B5	0	0	3	9	3	2	10	16.0
CR_P1_B6	2	0	6	4	2	0	NA	17.3
CR_P1_B7	2	6	2	15	4	0	0	32.3
CR_P1_B8	3	0	3	5	3	1	0	24.8
CR_P2_B1	2	2	0	5	12	6	20	6.3
CR_P2_B10	3	5	1	1	23	6	50	5.5
CR_P2_B11	3	7	0	0	16	2	15	14.8
CR_P2_B12	3	7	0	2	20	1	0	17.5
CR_P2_B13	9	2	3	24	15	1	0	14.8
CR_P2_B14	1	1	0	8	5	2	NA	16.0
CR_P2_B2	5	19	1	15	11	5	NA	11.0
CR_P2_B3	3	8	0	8	9	4	NA	16.5
CR_P2_B4	0	6	0	3	6	2	0	8.8
CR_P2_B5	3	12	0	2	6	4	NA	9.3
CR_P2_B6	1	8	1	11	12	0	0	6.0
CR_P2_B7	3	8	0	19	20	13	0	7.3
CR_P2_B8	1	11	0	32	9	1	0	10.8
CR_P2_B9	1	4	1	6	9	13	10	8.0
CR_P3_B1	0	11	1	7	26	2	NA	12.0
CR_P3_B2	1	2	1	11	10	9	5	15.5
CR_P3_B3	0	4	0	1	11	0	0	15.5
CR_P3_B4	1	14	1	53	9	0	0	16.7
CR_P3_B5	1	4	0	5	7	0	30	15.5
CR_P3_B6	1	4	1	16	12	0	0	15.8
CR_P3_B7	1	1	0	18	5	1	0	22.5
CR_P3_B8	1	5	0	5	9	0	NA	8.8
CR_P4_B1	3	0	1	45	10	5	0	14.8
CR_P4_B2	2	12	0	26	15	10	0	16.0
CR_P4_B3	3	3	0	14	17	6	NA	15.0
CR_P4_B4	1	3	1	4	13	1	NA	18.8
CR_P4_B5	2	4	0	19	17	7	5	11.5
CR_P5_B1	2	3	1	19	9	0	0	15.5
CR_P5_B10	46	3	0	1	20	0	0	20.0
CR_P5_B2	1	5	0	21	14	0	NA	17.5
CR_P5_B3	9	6	1	11	18	0	0	17.0
CR_P5_B4	4	11	0	9	17	0	45	18.0
CR_P5_B5	7	2	0	23	10	3	20	23.5
CR_P5_B6	2	2	0	39	13	5	0	20.3
CR_P5_B7	1	1	0	24	18	0	0	23.0
CR_P5_B8	1	2	0	52	8	3	0	19.8
CR_P5_B9	2	3	0	9	9	2	90	19.0
CR_P6_B1	2	3	1	12	5	0	0	26.3
CR_P6_B10	1	4	0	11	11	1	0	8.5
CR_P6_B2	0	2	1	1	6	0	0	29.8
CR_P6_B3	9	1	7	8	2	0	10	22.8
CR_P6_B4	2	5	5	7	7	0	0	23.0
CR_P6_B5	3	0	0	5	8	1	0	18.0
CR_P6_B6	0	5	1	1	7	0	25	23.3
CR_P6_B7	3	7	0	0	24	0	0	8.0
CR_P6_B8	1	4	0	8	10	0	0	7.5
CR_P6_B9	1	2	0	8	9	0	0	7.8
CR_P7_B1	3	6	0	8	4	2	10	28.8
CR_P7_B2	0	8	0	21	2	0	25	26.3
CR_P7_B3	1	3	1	10	6	0	NA	24.5
CR_P7_B4	5	7	0	25	8	0	0	26.8
CR_P7_B5	1	5	1	3	8	0	0	24.5
CR_P7_B6	1	1	4	4	1	1	NA	24.5
CR_P7_B7	3	5	0	2	8	1	50	22.3
CR_P7_B8	2	3	0	12	8	3	30	23.8
CR_P8_B1	1	1	0	11	6	7	5	12.0
CR_P8_B2	2	2	0	9	8	0	NA	14.8
CR_P8_B3	0	0	0	8	4	0	100	4.5
CR_P8_B4	2	2	0	18	15	0	10	8.5
CR_P9_B1	2	4	0	27	20	4	NA	9.3
CR_P9_B2	2	12	0	7	17	1	2	10.5
CR_P9_B3	1	2	1	10	11	9	NA	10.0
CR_P9_B4	2	5	0	16	20	1	NA	8.0
CR_P9_B5	3	7	0	8	3	0	60	6.8
CR_P9_B6	1	7	0	1	9	5	NA	7.0
CR_P9_B7	0	12	0	11	11	1	10	8.0
CR_P9_B8	2	6	0	11	9	6	NA	6.0

## 2 Experimental design, materials and methods

The experimental design is described in details in [1]. Below, we developed the calculation of total plant species richness and the implementation of the statistical models used to quantitatively estimate the effect of total plant species richness on arthropod groups’ abundance and corm damage.

### 2.1 Total plant species richness calculation

In [1], we estimated low stratum (plant height at top of the crown <1.5 m) and high stratum (plant height at top of the crown ≥1.5 m) plant species richness separately, and using different methods. In the present article, we calculated the total plant species richness as the sum of low- and high-stratum plant species richness.

### 2.2 Effect of total plant species richness on arthropod groups’ abundance and corm damage

Fig. 1 displays quantitatively the services or disservices associated with pest regulation provided by plant species richness, i.e. the effect of total plant species richness (sum of low- and high-stratum plant species richness) i) on adult weevil abundance and weevil corm damage score, (ii) on the abundance of herbivore and detritivore ground-dwelling arthropods, and iii) on the abundance of potential natural enemies (omnivorous ants, non-ant omnivores and predators). To account for uncontrolled field-scale effects, we added field identity as a random intercept effect [2]. R script used to obtain the Fig. 1 is provided in Appendix B.

## Appendix A Supplementary material

Supplementary material: The non-filtered dataset associated with this article and with [1] can be found in the online version.

.

Supplementary material: The R script associated with this article and with [1] can be found in the online version.
